# Transperitoneal Mini-Laparoscopic Pyeloplasty in Flank Position: A Safe Method for Infants and Young Adults

**DOI:** 10.3389/fsurg.2018.00032

**Published:** 2018-04-19

**Authors:** Beatriz Bañuelos Marco, Tom Florian Fuller, Frank Friedersdorff, Ricardo González, Anja Lingnau

**Affiliations:** Urology, Charité Universitätsmedizin Berlin, Berlin, Germany

**Keywords:** laparoscopic pyeloplasty, 3MM trocar, short instruments, flank position, laparoscopic pyeloplasty in children, infants

## Abstract

**Introduction and Objectives:**

Open dismembered pyeloplasty has been the gold standard treatment for ureteropelvic junction obstruction in children. Laparoscopic pyeloplasty (LP) is becoming a standard procedure, but its acceptance is slow. We report our method for minilaparoscopy (MLP) in children using a tansperitoneal approach with the patient in the lateral flank decubitus which we found technically advantageous.

**Materials and Methods:**

Retrospective review of the records of 52 children and adolescents up to 18 years of age who underwent transperitoneal MLP at our institution during March 2012–October 2017 A 5 mm trocar is placed for the camera at the site of the umblicus by open technique, two 3 mm trocars placed in the upper and lower quadrants of the abdomen. No additional ports were necessary. 20cm long, 3-mm-diameter instruments are used. Few cases needed percutaneous fixation of the pelvis. The anastomosis is performed with 5–0 or 6–0 Polyglecaprone 25 (Monocryl®) with 13 mm half circle needle (TF plus) suture cut to 12–14 cm length and introduced through the 5-mm port. Needles are removed through the 3-mm port under direct vision.

**Results:**

Fifty-two children (53 renal units) with a mean age of 82 months (range 3.5–204), a mean weight of 24,35 kg (range 7–57), and a mean follow-up of 20,44 months (6–60). Nine children were younger than 12 months, and 14 were ≤10kg. Six patients were >50kg. The mean of preoperative grade of dilatation was III (SFU scale) and postoperatively improved to SFU 0,60 (0–2). In 50 (94,3%) of the cases, there was complete resolution of hydronephrosis. There was no conversions to open surgery. Three patients suffered complications Clavien-Dindo Classification IIIb, 2 omental prolapses through a port site in two children which required general anaesthesia and one percutaneous drainage due to a leakage. No reinterventions related to stent complications or obstruction were found. Mean hospital stay was 4,69 (3–14) days.

**Conclusions:**

The method of mini LP described here has proven efficient and safe. Weight appeared not to be limitation for both groups ≤10 and >50 kg.

## Introduction

Open surgical dismembered pyeloplasty (Anderson-Hynes procedure) has long been the gold standard treatment for ureteropelvic junction obstruction (UPJ) in children (1). Laparoscopic pyeloplasty (LP) is becoming a standard procedure, but its acceptance is slow because of the difficulties in mastering the technique in particular intracorporeal suturing (2, 3). In children, the main demonstrable advantage of LP is cosmetic. Retroperitoneoscopy is preferred by some but the need to use a 10 mm port to create the space somewhat diminishes this advantage (4, 5). Also with the retroperitoneoscopic approach, the working space is limited, particularly in small children. For these reasons, there is a trend to prefer robotic assisted laparoscopic pyelopasty (RALP) because of the shorter learning curve (6). However, RALP is cosmetically inferior to an LP done with 3 mm working ports and its cost efficiency is debatable (7).

Most of the centres using minilaparoscopy (3 mm diameter, 20 cm length) in children use a transperitoneal approach (93,5%) (8). In most cases this approach is done with the child in the supine position (9). Here we report our method for mini LP using a tansperitoneal approach with the patient in the lateral flank decubitus as is often used for OP which we found technically advantageous. Our results in infants, children and adolescents are reported.

## Materials and Methods

We reviewed retrospectively the records of children and adolescents up to 18 years of age who underwent transperitoneal minilaparoscopic pyeloplasty (MLP) at our institution from March 2012 until October 2017, both included.

Patient demographics, weight, form of presentation, and outcomes were recorded. Pre- and postoperative imaging studies were reviewed. Indications for MLP included: Progressively increasing hydronephrosis detected pre- or neonatally, worsening or *de novo* appearance of symptoms (mainly abdominal pain), repeated urinary tract infection (UTI), decreased differential function in renogram (40% or less), or repeatedly obstructed curves on the diuresis renogram (2). Preoperative evaluation was assessed by the grading of hydronephrosis following the SFU classification (10) and the anterior posterior pelvic diameter was measured on transverse posterior views on ultrasonography. In 45 patients MAG3 diuretic renogram was performed. Sixteen patients underwent MRI to accurate the diagnosis, three of them due to the additional presence of nephrolithiasis. The presence of crossing vessels was confirmed intraoperatively in 22 patients.

### Method

**Figure 1:**
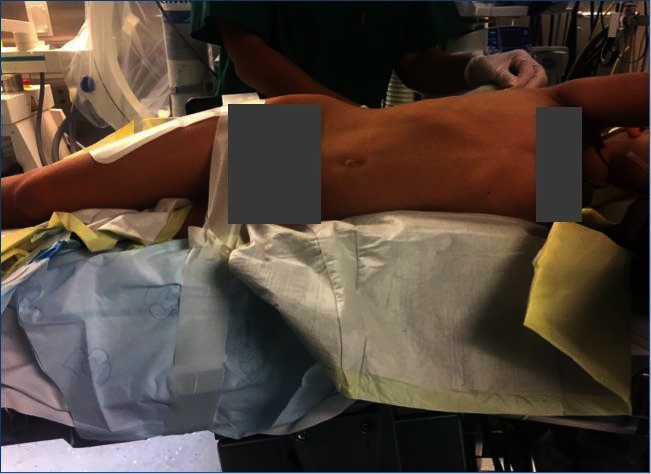
Flank position.

Under general anaesthesia cystoscopy and retrograde placement of double J stent were performed in most cases. However some patients a retrograde stent had been placed preoperatively and others had antegrade placement of the stent (see Table 3). A retrograde uretero-pyelogram was performed as necessary. An indwelling bladder catheter was left inside to fill the pelvis if necessary. The patient was then placed in lateral flank position with the side to be operated up. All pressure points were protected with pads, and the patient was fixed to the table with cloth tape, allowing the table to be rotated during the surgery. The surgeon and the camera were placed in front of the patient (Figure 1).

**Figure 2:**
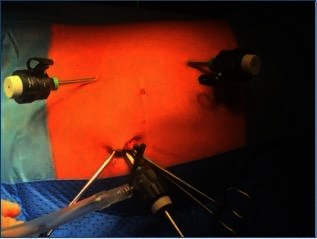
Trocars placement.

First a 5 mm trocar was placed for the camera at the site of the umbilicus by using open technique. Pneumoperitoneum was created and two 3 mm trocars were placed more laterally in the upper and lower quadrants of the abdomen (Figure 2). Additional ports were not needed in any patient. Twenty-centimetre-long, 3-mm-diameter instruments were used. Very few cases needed of percutaneous fixation of the pelvis using a 4/0 monocryl.

**Figure 3:**
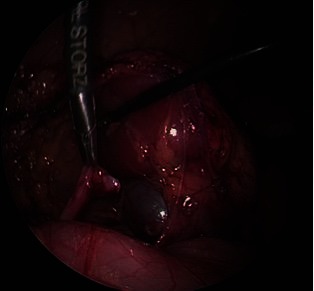
Dissection of the renal pelvis.

**Figure 4:**
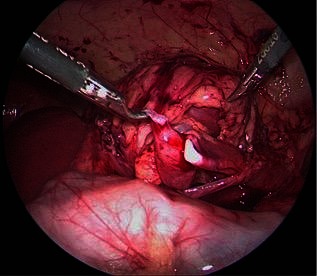
Dissection of the renal pelvis and spatulation of the ureter.

**Figure 5:**
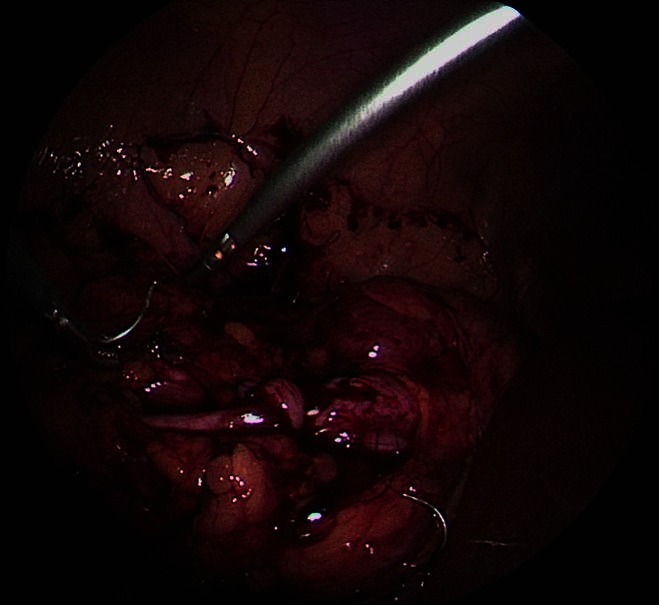
Anastomosis of the renal pelvis.

The UPJ was exposed after de colonic mobilisation (retrocolonic approach) in left sided cases. The hepatic flexure of the colon was mobilised for a right-sided approach as needed. Liver mobilization is rarely necessary given the position of the patient. Filling the bladder facilitates the dissection of the renal pelvis. Figures 3 and 4. Following the resection of the stenosed segment and spatulation of the ureter along its lateral aspect, 2 sutures are placed and tied in the lower corner of the anastomosis. Figure 5. The posterior suture line is done first followed by the anterior suture line. The anastomosis is performed with 5–0 or 6–0 Polyglecaprone 25 (Monocryl®) with 13 mm half circle needle (TF plus) suture cut to 12–14 cm in length and introduced through the 5 mm port. The needles were removed through the 3 mm port under direct vision.

## Results

Fifty two consecutive children (53 renal units) (15 females) with a mean age of 82 months (range 3.5–204), a mean weight of 24.35 kg (range 7–57), and a mean follow-up of 20,44 months (6–60) underwent laparoscopic pyeloplasty in our centre from March 2012 until October 2017 and included in this report. Nine children were younger than 12 months, and 14 were ≤10 kg. (Age Distribution is described in Table 1). The form of presentation included hydronephrosis diagnosed prenatally and confirmed postnatally, pain, urinary tract infections, hypertension, deteriorated renal function (elevation of creatinine). (Disease presentation is described in Table 2). One patient had bilateral methachronous pyeloplasties, five patients had previous renal surgery, and three patients suffered nephrolithiasis additionally.

**Table 1:** Age Distribution in 52 patients (53 renal units).

0–11 months	9
12–24 months	7
25–180 months	32
>180 months	4

Mean 84 months, range (3,5-204).

**Table 2:** Disease presentation at the time of Surgery.

Antenatal Hydronephrosis (n=)	11
Pain (n=)	22
Infection (n=)	10
Hypertension (n=)	1
GFR <90 (n=)	4
Renal Function <15% of the affected renal unit provided by MAG3 (n=)	5

The mean of preoperative grade of dilatation was III (SFU scale) and postoperatively improved to SFU 0.60 (0–2). In 50 (94.3%) of the cases, there was complete resolution of hydronephrosis (grades 0–1). Three patients didn’t complete a minimum of 6 months follow up and therefore they are excluded from the correspondent mean. Nevertheless, all of them achieved a three months postoperative control and are included in the results concerning our laparoscopic pyeloplasty technique. There were no conversions to open surgery. Three patients suffered complications Clavien-Dindo Classification IIIb. Two patients suffered an omental prolapse through a port site after drainage removal, which were reduced under general anaesthesia. One patient required placement of a percutaneous drain due to a urine leak. There were no re-interventions related to stent complications or recurrent obstruction. One patient due to intraoperative acidosis required an intensive care unit bed for bicarbonate correction regime. Despite the increasing acceptance in urology, the Clavien–Dindo classification has never been widely validated for urological procedures and is not a reliable tool for use in pediatric urology (11, 12). Mean hospital stay was 4.69 (3–14) days (See Table 3).

**Table 3:** Pre-, intra- and postoperative factors related to the surgical intervention.

Follow up time in months. Mean (range)		20,44 (6–60)
Complications patients (percent)		5 (9.4%)
Clavien (n=)	I	1
	IIIb	3
Intensive care bed postoperative (one night for bicarbonate correction)		1
Conversion		None
Hospital stay (days) for operation performed		4.69
Preoperative retrograde DJ-Stent. (n=)		10
Intraoperative retrograde DJ-Stent. (n=)		23
Intraoperative antegrade DJ-Stent. (n=)		20

Results are shown for the total number of pyeloplasties performed.

In the 4 patients with decreased renal function the creatinine level normalized after MLP. In one patient presenting with hypertension and bilateral UPJ obstruction the hypertension resolved after the first MLP. All the patients presenting in the renography less than 15% of the split renal function of the affected kidney showed a recovering function, resolution of the symptoms (hypertension, infection or pain) and/or normalisation of the creatinine value.

## Discussion

Our results demonstrate that mini-laparoscopic pyeloplasty is safe for infants and also for young adults. Weight appeared not to be a limitation. The results for patients ≤10 and >50 kg were comparable. Our approach allowed the use of 3 mm ports and short instruments in infants, children and adolescents or young adults up to 18 years old with the aim of facilitating the operation leaving minimal scars. Complications such as omental prolapse, through a port site after drainage removal in children require unfortunately general anaesthesia to be solved. Based on our experience, considering the low rate of complications which required a postoperative percutaneous drainage (one out of fifty-three operations) and the known complications related to the use of a surgical drain, we recommend its prophylactic use only in complicated cases (i.e., nephrolithiasis).

Open surgical dismembered pyeloplasty (Anderson-Hynes procedure) remains as the predominant treatment modality for ureteropelvic junction obstruction (UPJ) in children (1). Sukumurar et al. (3) analysed the national trends of the various treatments for paediatric ureteropelvic junction obstruction (UPJO), over a total of 35,275 children. Although there has been a substantial increase in minimally invasive pyeloplasty after 2007, while RALP continues to increase its rates, those from LP appear to be stable since 2003.

Many studies (6, 8) demonstrate that the complication rates are low in the three approaches (OP, RALP and LP) and validates their safety and feasibility. Furthermore Chan et al. (6) proved that the benefits of laparoscopy are not only limited to older children but also apply for infants. However, they insist on the application of robotics.

 The use of the RALP in paediatric urology is becoming more popular with the aim of overcoming the technical difficulties associated with the limited small space of the abdominal cavity in infants, or to the size of the instruments used through 3 and 5 mm ports, such as their length. The results of a recent study involving 6 surgeons, all of them with more than 5 years experience performing 62 procedures in a total of 60 patients showed a 91% of resolution of the hydronephrosis and 11% rate of complications (13). However, we must take in account the cost-efficiency of the RALP; including the need for an operation room at least 60 m^2^, back-up equipment in case of failures (14) and the cosmetic results related to larger ports compared to the MLP.

Up to the present time there is a lack of standardized technique amongst institutions both for the laparoscopic pyeloplasty techniques (retroperitoneoscopy and transperitoneal approach) and the RALP in the paediatric patient.

Each method presents its advantages and inconveniences, and until now, none has shown clear superiority over the others. As the future goals of all the minimal invasive techniques should be minimizing the impact of the scars without losing safety and control, MLP offers a feasible technique with the most cosmetic advantages. The method of mini LP described here has proven efficient and safe. The flank position allows the use of short 3 mm instruments, with the attending advantage of easier suturing and minimal scars not only for infants under 12 months of age but also in patients over 50 kg of body weight.

Our study has several limitations because of its retrospective nature and the small number of patients belonging to different age groups. Nevertheless, in our experience the MLP in the flank position is a feasible, safe and successful approach not only for children and for infants, but also can be extended to young adults safely.

## Ethics Statement

This study was carried out in accordance with the recommendations of Ethik- Kommission des Landes Berlin (Landesamt für Gesundheit und Soziales Berlin), with written informed consent from all subjects. All subjects gave written informed consent in accordance with the Declaration of Helsinki. Retrospectiv studies are exempt from Protocol Approval following the guidelines of the Ethik- Kommission des Landes Berlin. The confidentiality of all the subjects was protected.

All the patients received the treatment according to the standard care recommended for this urological pathology. All the patients included in the study met the criteria to undergo a surgical treatment; this being Anderson Hynes dismembered pyeloplasty the currently Gold Standard Therapy recommended in the European Guidelines of Urology For retrospective studies the Berlin State Ethics Committee (Geschäftsstelle der Ethik-Kommission des Landes Berlin) doesn't require an approval of the clinic ethical committee.

The character of this study is a retrospective evaluation of the Standard Treatment performed to our patients with Pelvic-Ureteric Junction Obstruction.

## Author Contributions

BM performed operations, data collection and writing manuscript, RG reviewed the manuscript, FF assisted with data collection, AL performed operations and supervision of all, assisted with data collection and preparation manuscript. TF contributed to the development of the technique during thefirst two years of the patient's series, revisinig the final version of the manuscript after the necessary changes

## Conflict of Interest Statement

The authors declare that the research was conducted in the absence of any commercial or financial relationships that could be construed as a potential conflict of interest.
